# Adaptive high microvascular resistance: a diagnostic dilemma in coronary physiology

**DOI:** 10.1093/ehjopen/oeaf073

**Published:** 2025-06-06

**Authors:** Ioannis Skalidis, Philippe Garot, Panagiotis Antiochos

**Affiliations:** Institut Cardiovasculaire Paris-Sud, Hôpital Jacques Cartier, 6 Avenue du Noyer Lambert, Ramsay-Santé, Massy 91300, France; Institut Cardiovasculaire Paris-Sud, Hôpital Jacques Cartier, 6 Avenue du Noyer Lambert, Ramsay-Santé, Massy 91300, France; Cardiology Department, Lausanne University Hospital, Rue du Bugnon 46, 1005 Lausanne, Switzerland

**Keywords:** Coronary Microvascular Dysfunction, Microvascular Resistance, ANOCA

**This commentary refers to “The impact of high microvascular resistance on coronary wave energetics depends on coronary microvascular functionality”, by A. Tas *et al.*, https://doi.org/10.1093/ehjopen/oeaf050**.

The recent publication by Tas *et al*.^1^ offers a compelling reevaluation of the diagnostic significance of elevated hyperaemic microvascular resistance (hMR) in patients with unobstructed coronary arteries.^1^ By leveraging wave intensity analysis (WIA), the authors investigate how hMR interacts with coronary microvascular functionality, revealing that high hMR in the presence of preserved coronary flow reserve (CFR) may reflect a physiological adaptation rather than a pathological state. This distinction, underscored by robust autoregulatory responses in the so-called ‘no CMD–high hMR’ endotype, challenges current diagnostic conventions that often equate high hMR with coronary microvascular dysfunction (CMD). The study thereby contributes a critical perspective to the ongoing effort to refine physiological criteria in the assessment of angina/ischaemia with non-obstructive coronary arteries (ANOCA/INOCA).

The findings are timely, particularly in light of growing recognition that diagnostic algorithms relying on fixed thresholds—such as CFR < 2.5 and hMR ≥ 2.5 mmHg·s·cm⁻^1^—may oversimplify the complex continuum of microvascular behaviour. Tas *et al*.^1^ demonstrate that vessels with elevated hMR but preserved CFR exhibit the most pronounced hyperaemic augmentation in accelerating wave energy proportion (AEP), along with high resistive reserve ratio (RRR) and microvascular resistance reserve (MRR). These features are suggestive not of dysfunction, but of preserved autoregulatory capacity and adaptive vascular tuning. The study thus supports a physiology-based framework over a purely threshold-based one.

Notably, the use of WIA in this context is innovative, offering mechanistic insights into cardiac-coronary coupling beyond conventional flow and pressure indices. However, as the authors acknowledge, certain aspects merit further exploration. One such area is the potential redefinition or stratification of diagnostic cut-offs. If high hMR can reflect either adaptive or pathological processes depending on CFR and AEP response, a more nuanced classification may be needed in future guidelines—possibly incorporating dynamic indices such as ΔAEP.

Another noteworthy point is the study’s limited female representation. The authors report that women did not exhibit significant differences in wave energetics between CMD and non-CMD groups, which contrast with prior studies highlighting distinct sex-based patterns in microvascular disease. Whether this reflects physiological variance or is attributable to statistical underpowering remains an open question. Future studies designed with balanced sex representation are essential to assess the generalisability of the adaptive high hMR phenotype.

Finally, although AEP and other WIA-derived parameters were not strongly correlated with CFR, RRR, or MRR across the full cohort, their prognostic utility remains to be established. Whether WIA can help identify subgroups with differing risk profiles or treatment responses—particularly among patients with ambiguous physiological profiles—warrants further longitudinal investigation.

In conclusion, this study makes an important contribution by decoupling elevated hMR from CMD when CFR is preserved and calls for a more nuanced, physiology-informed interpretation of coronary resistance data. The findings not only refine our understanding of coronary microvascular behaviour but also underscore the importance of integrating dynamic physiological assessments into diagnostic frameworks. Prospective studies with diverse populations and outcome data will be essential to validate and operationalize these insights in clinical practice.
